# Nitrogen K-edge X-ray adsorption near-edge structure spectroscopy of chemically adsorbed ammonia gas on clay minerals and the ^15^N/^14^N-nitrogen isotopic fractionation

**DOI:** 10.1007/s44211-023-00503-5

**Published:** 2024-02-05

**Authors:** Haruna Sugahara, Toshihiro Yoshimura, Yusuke Tamenori, Yoshinori Takano, Nanako O. Ogawa, Yoshito Chikaraishi, Naohiko Ohkouchi

**Affiliations:** 1 Biogeochemistry Research Center (BGC), Japan Agency for Marine-Earth Science and Technology (JAMSTEC), Natsushima, Yokosuka, Kanagawa 237-0061 Japan; 2 Institute of Space and Astronautical Science (ISAS), Japan Aerospace Exploration Agency (JAXA), 3-1-1 Yoshinodai, Chuo-Ku, Sagamihara, Kanagawa 252-5210 Japan; 3 Japan Synchrotron Radiation Research Institute, SPring-8, 1-1-1 Kouto, Sayo, Hyogo 679-5198 Japan; 4 Organization for Research Promotion, Tokyo Metropolitan University, Minami-Osawa, 1-1, Hachioji, Tokyo 192-0397 Japan; 5 Institute of Low Temperature Science, Hokkaido University, Sapporo, Hokkaido 060-0819 Japan

**Keywords:** Ammonia, Nitrogen isotopic fractionation, XANES, Adsorption, Clay mineral

## Abstract

Ammonia (NH_3_) is a simple and essential nitrogen carrier in the universe. Its adsorption on mineral surfaces is an important step in the synthesis of nitrogenous organic molecules in extraterrestrial environments. The nitrogen isotopic ratios provide a useful tool for understanding the formation processes of N-bearing molecules. In this study, adsorption experiments were conducted using gaseous NH_3_ and representative clay minerals. The strongly adsorbed NH_3_ was ^15^N-enriched in a state of chemical equilibrium between the adsorption and desorption on the siliceous host surface. The nitrogen K-edge X-ray adsorption near-edge structure spectroscopy study revealed that these initial ammonia gases were chemically adsorbed as ammonium ions (NH_4_^+^) on clay minerals.

**Graphical abstract:**

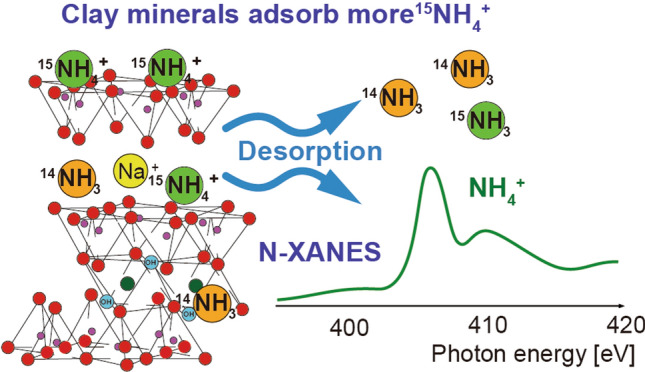

## Introduction

Nitrogen (N) is an abundant element in the universe and forms various organic molecules by bonding with carbon (C), hydrogen (H), and oxygen (O). Ammonia (NH_3_), the primordial N, is the major N carrier in interstellar molecular clouds [1–3], as well as in the solar system bodies. Because of its high chemical reactivity, NH_3_ is considered as the starting molecule for various N-containing molecules. Interstellar dust surfaces are important reaction sites for the formation of organic molecules in photochemical reactions. The adsorption of NH_3_ on interstellar dust surfaces is the first step in their chemical reactions on interstellar dust surfaces.

N has two stable isotopes, ^14^N and ^15^N. The isotope ratios (δ^15^N, ‰: per mille deviation of the ^15^N/^14^N ratio from the Earth’s atmosphere) of molecules provides information on the environment and reaction processes that the molecules have experienced. Solar system bodies exhibit a wide range of the δ^15^N values. For instance, Sun and Jupiter have the δ^15^N values of − 350‰, whereas solid planets such as Earth, Venus, and Mars have the δ^15^N values of 0‰. Meanwhile, more primitive solar system materials, such as comets and chondrites, have the elevated δ^15^N values of >  + 1000‰ for comets [4], and <  + 200‰ for carbonaceous chondrites as bulk values [5]. In addition, there are large isotopic anomalies (< + 5000‰) in chondrites and cometary dust with significant local enrichment of heavy isotopes, which are called hotspots [6–8]. The ^15^N enrichment is considered to have originated in cold interstellar molecular clouds [9, 10] and star-forming regions [11, 12]. Several mechanisms of nitrogen isotopic fractionation in the interstellar environments have been proposed such as ion–molecule exchange reactions between N atoms and N-containing ions [13, 14] and isotope-selective UV photodissociation of N_2_ and formation of ^15^N-enriched NH_3_ ice [9, 12, 15]. However, the relevant mechanisms are not well understood, yet.

The isotopic fractionation of N during the adsorption and desorption of NH_3_ gas on solid particle surfaces has been previously reported by Sugahara et al. [16]. Laboratory experiments in which NH_3_ gas (δ^15^N =  + 27.0‰) was adsorbed on two Japan Clay Science Society (JCSS) standard siliceous clay minerals (montmorillonite and saponite) revealed a negative correlation between the N isotope ratios of the clay minerals and the adsorption rate of NH_3_, with a large N isotopic fractionation greater than + 50‰ [16]. The study further verified the δ^15^N changes during vacuum-induced desorption, revealing that the δ^15^N values of the N remaining on the clay sample decreased after a brief increase and finally settled at a certain value. These facts suggest that there are at least two chemical forms for the adsorption of NH_3_: one is easily desorbed under vacuum, and the other is strongly adsorbed on clay minerals. The former is in the form of an NH_3_ molecule, which are physically trapped in the interlayer space of clay minerals and replacements of the water molecules coordinated to interlayer cations, and the coordination to the Lewis acid sites and silanols. The latter is in the form of ammonium ion (NH_4_^+^), which were ionized by reacting with strong Brønsted acid sites such as surface-adsorbed water or interlayer water [17, 19].

X-ray absorption near-edge structure (XANES) is a molecular-scale analytical technique that determines the chemical environment from the electronic and structural information of a target element [20]. The chemical properties of specific elements can be studied, and information regarding the geometric arrangement of such elements of interest can be obtained. Because the chemical environment of N is fundamental to the biogenic cycle on Earth, N *K*-edge XANES has been applied to identify the form in which N is present in organic and inorganic composite materials [21], such as soils and sediments [22, 23]. The substitution of NH_4_^+^ into clay minerals and their XANES spectra have been reported by Leinweber et al. [22]; however, NH_3_ gas-adsorbed clay has not yet been verified.

In this study, XANES analysis was performed at the N-targeting *K*-edge to clarify the relationship between the N isotopic anomalies and the adsorption mechanism of NH_3_. Because the adsorption of NH_3_ was extremely small (0.1–1.3 wt%), determining the chemical form of N nondestructively and nonextractively in a laboratory experiment is impossible. In this regard, the partial fluorescence yield (PFY) method, a type of XANES, can be applied for high-sensitivity analyses on the order of ppm [24–26]. Thus, PFY-based XANES analysis was performed on four types of clay mineral samples (montmorillonite, saponite, dickite, and kaolinite) with adsorbed with NH_3_ molecules to determine the differences between their adsorption spectral characteristics and N chemical forms.

## Materials and methods

Adsorption experiments on NH_3_ gas were conducted using reference clay samples of JCSS-3101b montmorillonite [(Na,Ca)_0.33_(Al,Mg)_2_Si_4_O_10_(OH)_2_·*n*H_2_O], JCSS-3501 saponite [Ca_0.25_(Mg,Fe)_3_((Si,Al)_4_O_10_)(OH)_2_·*n*H_2_O], JCSS-1301 dickite [Al_2_Si_2_O_5_(OH)_4_], and JCSS-1101b kaolinite [Al_4_Si_4_O_10_(OH)_8_]. The surface area data measured using the N-adsorption Brunauer–Emmett–Teller (BET) method were compiled with the previous results [16] (Table 1).

**Table 1:** Types of clay minerals used in the study

JCSS reference ID	Mineralogy		AIPEA type	BET SSA (m^2^/g)	Median diameter of the particle (µm)*	CEC (meq/100g)**
JCSS-3101b	Montmorillonite	2:1	Dioctahedral smectite	10.8	1.312	114.4
JCSS-3501	Saponite	2:1	Trioctahedral smectite	201	0.034	99.7
JCSS-1301	Dickite	1:1	Kaolinite	4.7	7.521	2.8
JCSS-1101b	Kaolinite	1:1	Kaolinite	20.2	10.806	3.9

SSA denotes the specific surface area. CEC denotes the cation exchange capacity and indicates the total amount of cations that can be retained in clay minerals. The smectite group minerals (montmorillonite and saponite) had a large CEC, whereas the kaolinite group (dickite and kaolinite) had a small CEC

^*^Data obtained from Miyawaki et al. [49]

^**^CEC data for montmorillonite from Oshima et al. [50], those for saponite from Takagi et al. [51], those for dickite from Tone et al. [52], and those for kaolinite from Tone et al. [53]

**Fig. 1:**
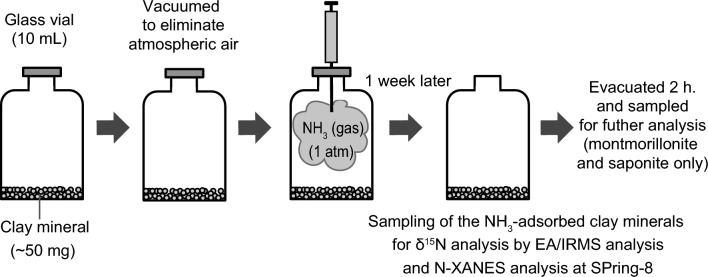
Schematic illustration of the NH_3_-adsorption experiments conducted in this study. Individual clay minerals (~ 50 mg each) were placed in 10-ml glass vials. The glass vials were evacuated to eliminate atmospheric air. NH_3_ gas was then introduced into the glass vials, which were adjusted to 1 atm. After stabilization for a week, the glass vials were opened, and portions of the NH_3_-adsorbed clay minerals were sampled for δ^15^N analysis through EA/IRMS and N-XANES analysis at SPring-8. Montmorillonite and saponite were further evacuated for 2 h and sampled for the same analysis

The details of the adsorption experiments and N isotope analysis were described by Sugahara et al. [16]. Briefly, the individual clay minerals (50 mg each) were placed in 10-mL glass vials (Fig. 1). The clay minerals were preheated at 110 °C for more than a week to eliminate adsorbed water (Fig. 1). After the glass vials were sealed, atmospheric gas in the glass vials was evacuated. NH_3_ gas with a known δ^15^N value of + 27.0‰ was then introduced into the vials. The gas pressure was set at ~ 1 atm. The glass vials were kept at room temperature for one week. A sensitivity-improved elemental analyzer connected to an isotope-ratio mass spectrometer (nano-EA/IRMS) was used to determine the N content and their δ^15^N values [16, 27]. The nitrogen isotope values are expressed as conventional δ notation relative to atmospheric N_2_ (air). An authentic standard IAEA-N-2 (ammonium sulfate, + 20.3‰) and an interlaboratory calibrated standard BG-T (L-tyrosine, + 8.74‰, [28]) were used to calibrate the δ^15^N values. The δ^15^N analysis of standards and samples was performed in quantities of 18–250 nmol (0.3–3.4µg) nitrogen, and the analysis error estimated from repeated analysis of IAEA-N-2 was less than ± 0.5‰ (n = 5). After the initial analysis, part of the residual samples of montmorillonite and saponite were evacuated for 2 h and those of dickite and kaolinite for 1 h to examine the change in the adsorbed NH_3_ by this process. The evacuated samples were also analyzed using nano-EA/IRMS.

## N-targeting *K*-edge XANES analysis

**Fig. 2:**
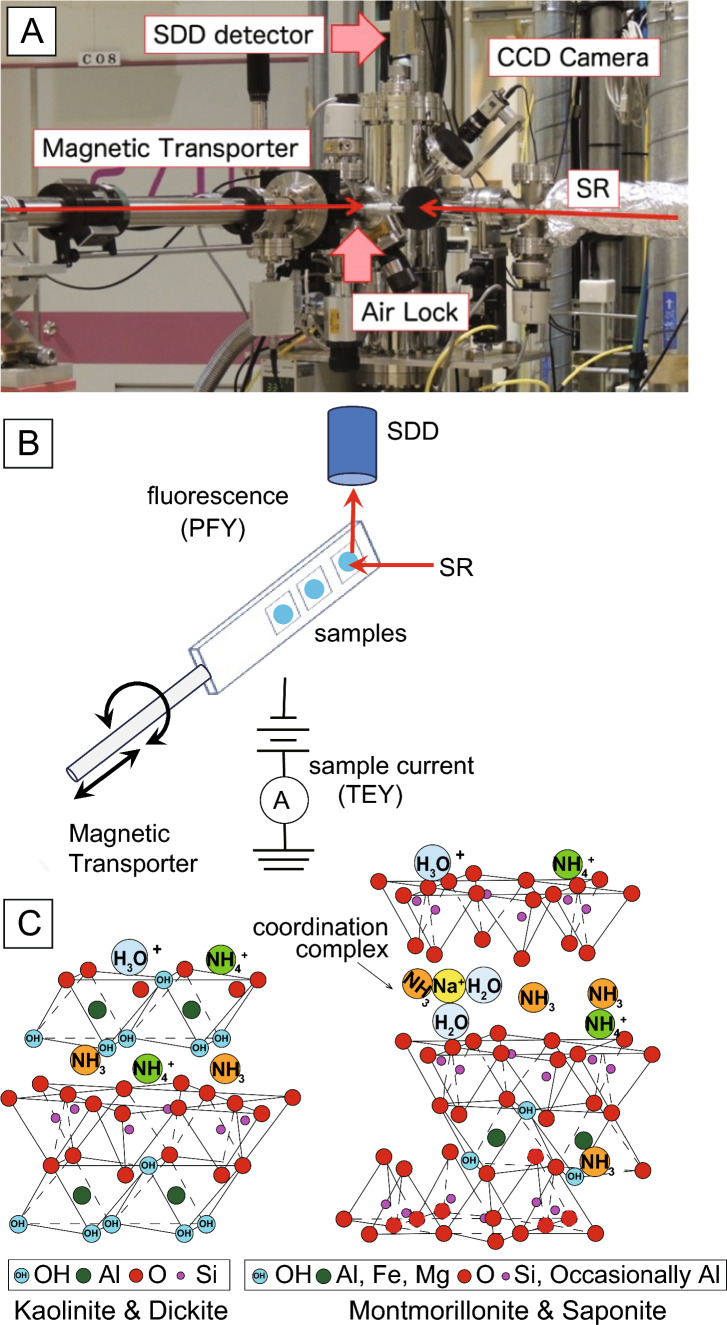
**A** Photograph of the soft X-ray beamline facility of BL27SU at SPring-8. **B** Schematic illustration of the analytical setup for the nitrogen *K*-edge XANES measurement. **C** Mineral structures of clay minerals and the possible adsorption states of ammonia. *SDD* silicon drift detector, *CCD* charge-coupled device, *SR* synchrotron radiation, *PFY* partial fluorescence yield; and *TEY* total electron yield

The XANES measurements were performed at the c-branch of the Soft X-ray Photochemistry Beamline (BL27SU) at SPring-8 (Fig. 2). The light source was radiation from a figure-eight undulator that produced a linearly polarized photon beam [29]. The photon beam was dispersed using a soft X-ray monochromator with a plane grating with different line spacings [30]. XANES spectra were obtained by scanning the undulator gap to maximize the intensity of the incident soft X-ray and scanning the monochromator to keep the resolving power constant. Measurements were taken by scanning the widths of the entrance and exit slits. The photon energy resolution during measurements was set to 80 meV. The beam size at the focal point was circular with a diameter of 500 µm and a photon flux of 1 × 10^11^ Ph/s [31]. During the XANES measurements, the intensity of the incoming photon beam (*I*_*0*_) was monitored by measuring the drain current at the surface of the post-focusing mirror.

For the XANES measurements, powdered clay mineral standards adsorbed with NH_3_ were fixed to a sample holder with conductive double-sided carbon tape. The sample holder was fixed to a linearly rotating manipulator and placed in a vacuum chamber at 1 × 10^−5^ Pa. The spectra were recorded by applying the PFY method using a silicon drift detector (SDD) [32]. The materials used in this study are listed in Table 1. The energy range for the N *K*-edge XANES measurements was 395–415 eV, with an energy step of 0.1 eV and an acquisition time of 4 s. As finer spectral structures have been reported only for N_2_ gas [33], an additional energy step of 0.01 eV was set in the 399.5–402.0-eV interval for additional analysis to confirm the presence of N_2_ gas. Data analysis for background removal and qualitative analysis of the XANES spectra were normalized to variations in the primary X-ray intensity (*I*_*0*_). Linear pre-edges were removed for each spectrum and the data were normalized using the edge jump height. Because of the low N concentration in the samples, PFY data were used owing to the high sensitivity of the measurement.

## Results and discussion

**Fig. 3:**
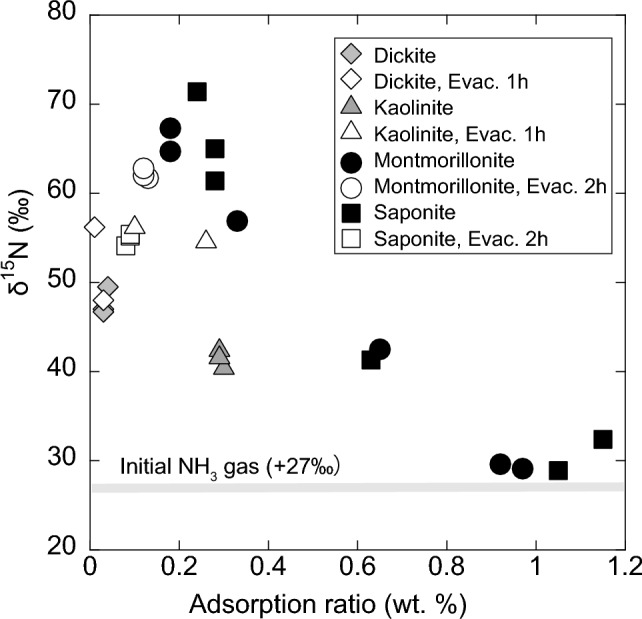
Variation in the nitrogen isotope ratios of ammonia gas adsorbed on clay minerals. The adsorption ratio of each clay mineral was normalized to the wt%. Filled circle, unfilled circle, filled square, unfilled square after Sugahara et al. [16]

Every sample showed the increased δ^15^N values compared to the initial δ^15^N value of the introduced NH_3_ gas (+27‰) (Figure 3 and Table 2). Dickite and kaolinite were plotted in the specific areas with lower adsorption ratios as shown in Figure 3. Dickite and kaolinite showed mean δ^15^N values of +47.7‰ and +41.8‰, with adsorption ratios of 0.03% and 0.29%, respectively. In contrast, our previous results showed that the nonevacuated samples of montmorillonite and saponite had variations in the δ^15^N values and their adsorption ratios indicating a negative correlation between them. Their δ^15^N values ranges from +28.9‰ to +71.4‰, whereas the adsorption ratios ranged from 0.18% to 1.15% [16]. These results demonstrated that the adsorption of NH_3_ caused the isotopic fractionation of N, and the adsorbed NH_3_ was enriched in ^15^N.

**Table 2:** Raw profiles of nitrogen adsorption (wt%) and nitrogen isotopic composition (δ^15^N, ‰ vs. Air) measured using nano-EA/IRMS

	δ^15^N (‰ air)	Adsorption ratio of NH_3_(wt.%)
Dickite	49.5	0.04
	47.0	0.03
	46.7	0.03
Dickite Evac. 1h	48.0	0.03
	56.2	0.01
Kaolinite	42.7	0.29
	40.7	0.30
	41.9	0.29
Kaolinite Evac. 1h	54.9	0.26
	56.5	0.10

The dataset for montmorillonite and saponite were shown in a previous report [16]

After the evacuation of the NH_3_-adsorbed samples, the evacuated (1 h) dickite and kaolinite were plotted in a specific area with the elevated δ^15^N values of +52.1‰ and +55.7‰, respectively, with slightly decreased adsorption ratios. This result is consistent with the evacuated (2 h) montmorillonite and saponite, which were plotted in similar areas with uniform values. The adsorption ratios were 0.12% for the 2-h-evacuated montmorillonite and 0.09% for the 2-h-evacuated saponite, which were between those of dickite and kaolinite. The mean δ^15^N values were +62.2‰ and +54.9‰, respectively. These observations implied that the light isotope (^14^N) was preferentially removed by evacuation and that the remaining NH_3_ was enriched in ^15^N and the remaining NH_3_ existed in only one adsorption form.

Variations in the adsorption ratios and δ^15^N values, which were observed in nonevacuated montmorillonite and saponite, were due to the existence of exchangeable cations (e.g., Na^+^ and Ca^2+^) and interlayer water in the interlayer space of these clay minerals, whereas dickite and kaolinite did not. According to the Fourier Transform Infrared (FTIR) Spectroscopic study of montmorillonite and saponite, which were exposed to gaseous NH_3_, two different acidities of these clay minerals: Lewis acid and Brønsted acid, are keys to understand the adsorption mechanisms of NH_3_ [17, 19]. In the interaction with the Lewis acid site (e.g., aluminum ions), NH_3_ adsorbed as NH_3_ molecules [17, 19]. The hydrogen bonding with silanols also has a contribution in this adsorption mechanism [17, 19]. On the other hand, the reaction with the Brønsted acid site (e.g., interlayer water molecules) produce ammonium ions (NH_4_^+^) [17, 19]. This ionized NH_4_^+^ also further connect with NH_3_ molecules via hydrogen bonding [17, 34]. The acidity of the Lewis acid sites and silanols are week and the adsorbed NH_3_ are easily evacuated [17]. On the contrary, NH_3_ adsorbed on montmorillonite and saponite after evacuation, as well as on dickite and kaolinite, was strongly adsorbed on the mineral surfaces. They are protonated at the Brønsted acid site to form NH_4_^+^ and chemisorbed on the mineral surface. The elevated δ^15^N values of the chemisorbed NH_4_^+^ represented N isotopic fractionation caused by the protonation and chemisorption processes of NH_3_, and they were different among clay minerals.

**Fig. 4:**
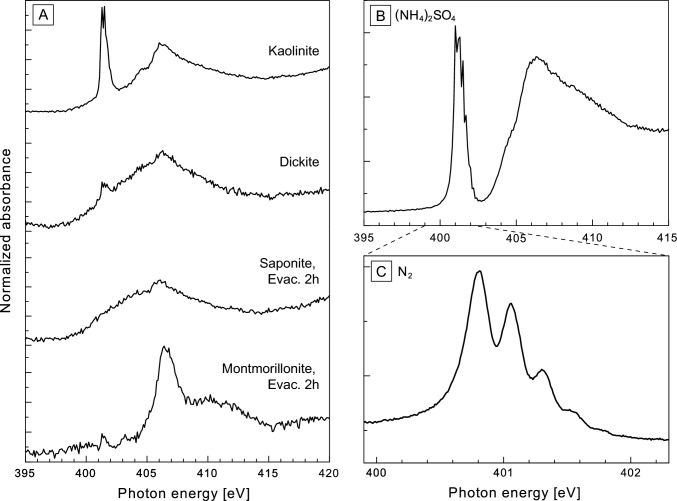
**A** Nitrogen *K*-edge XANES spectra of clay minerals adsorbed with NH_3_ gas and **B** ammonium sulfate powder. **C** 401-eV resonance characteristics of N_2_ measured with a high-resolution scan at 0.01-eV steps

The N *K*-edge XANES spectra obtained for NH_3_-adsorbed clay minerals are shown in Fig. 4. The N-XANES spectra were obtained for non-evacuated dickite and kaolinite and 2-h-evacuated montmorillonite and saponite. We also tried to measure the nonevacuated montmorillonite and saponite, but it was not succeeded. This was probably because gaseous NH_3_ easily escaped and/or decomposed by X-ray radiation. All NH_3_-adsorbed clay minerals exhibited similar N-XANES spectra. They had a relatively broad peak at 406 eV in common. Kaolinite also had a sharp peak at 401 eV. A weak 401-eV peak was also observed in dickite and 2-h-evacuated montmorillonite. In addition, 2-h-evacuated montmorillonites also had a broad feature around 410 eV.

Only a few N *K*-edge XANES studies have been conducted on solid soil samples [35, 36]. The spectra of bentonite and soil clay shared a prominent peak at 405.5 eV in the 1s → σ* region and a broad resonance feature of interlayer NH_4_^+^ near 410 eV. A previous study [22] reported the XANES spectra of montmorillonite (bentonite) substituted with NH_4_^+^ in the interlayer. This interlayer NH_4_^+^ has been proposed as a broad resonance at approximately 410 eV and is recognized in the spectra of fine and coarse soil samples [22]. Herein, the 410-eV interlayer NH_4_^+^-derived peak was more prominent in the 2-h-evacuated montmorillonite (Fig. 1). Smectite is generally a 2:1-type mineral with large amounts of exchangeable cations and water molecules in the interlayer and is a material with high viscosity, swelling, cation exchange capacity (CEC), and adsorption properties. The N *K*-edge XANES spectra of montmorillonite that reacted with NH_3_ gas showed that the adsorbed NH_3_ was chemisorbed as NH_4_^+^ between the layers after vacuum removal. In the kaolinite, dickite, and 2-h-evacuated saponite spectra, no distinct interlayer N peaks were observed, as in the 2-h-evacuated montmorillonite (Fig. 4).

Leinweber et al. [22] showed N *K*-edge XANES spectra for clay fractions in soil and identified three peaks between 398.8 and 401.0 eV. Based on the spectral characterization of reference compounds by Leinweber et al. [21], the peak at 398.8 eV and 399.8 eV were considered to be represented several nitrogeneous organic compounds such as nitrogen heterocycles and nitrile. The 401-eV could be also originated from some nitrogeneous organic compounds such as amino acids and nitrogen heterocycles. Ammonium salts also have 401-eV peak and could be the candidate. The peak at 405.5 eV, which was located on the higher energy side than these peaks, was derived from the 1s → σ* resonance of N-compounds and was considered to be a common feature in a wide variety of N-containing compounds. This 405.5-eV peak corresponds to the 407-eV peak in our NH_3_-adsorbed clay minerals. Meanwhile, the 401-eV peak is controversial because it is reported to the result of beam damage products for amino acids [37]. The distinct peak at 401 eV in kaolinite and the same small peak in the 2-h-evacuated montmorillonite and dickite are derived from N_2_. The presence of N_2_ can be easily distinguished that from of amine by measuring a high-resolution spectrum near 401 eV because a fine structure (N–N stretching vibration: ν = 235 meV) appears in the peak corresponding to the 1s → π* transition [33] (Fig. 4c). Our sample also confirmed the presence of N_2_ at ~ 401 eV using high-resolution measurements (Fig. 4c). This peak was not observed in the blank sample and thus was not due to residual N_2_. The appearance of the N_2_ signal in the soil can be explained by the photoinduced decomposition of NH_4_^+^ [38]. Moreover, the irradiation experiment of 150-eV photons on an NH_3_ ice film at 20 K demonstrated the formation of N_2_ by a series of photochemical reactions via intermediate radical species such as NH_2_, NH, N_2_H_4_, N_2_H_3_, and N_2_H_2_ [39]. Compared with their photon irradiation experiments [40], the photon intensity in our XANES measurements was very strong. In addition, the experimental temperature was higher (20 K vs. room temperature) in our experiments. Our experimental condition would work more for these photochemical reactions than their settings. Therefore, it is plausible that some NH_3_ and/or NH_4_^+^ adsorbed on clay minerals were decomposed through photoinduced decomposition by soft X-rays, and that this N_2_ derived from the decomposition of ammonium species (NH_3_, NH_4_^+^) was the origin of the 401-eV peak, which is indirect evidence for adsorbed NH_3_ and/or NH_4_. That N_2_ peak was very large for kaolinite, and the amount converted to N_2_ in the photochemical reaction seemed to vary depending on the structure of the clay mineral. In addition, the saponite peak exhibited a shoulder approximately 405 eV to the left of the main peak. This feature was also observed in kaolinite and dickite. NH_3_ molecules adsorbed on metal surfaces are known to have a broad peak around this region [40, 41]. Thus, this feature may indicate remaining NH_3_, which could exhibit been trapped in the interlayer space of the clay minerals.

The spectral similarity between bentonite loaded with interlayer NH_4_^+^ [22] and our NH_3_-adsorbed clay minerals implies that the N *K*-edge XANES spectra in our study show chemisorbed NH_4_^+^ in the interlayer space. This is consistent with the interpretation derived from the N isotopic results. Although a study has reported the N *K*-edge XANES spectra of interlayer NH_4_^+^ [22], there have been no reports on adsorbed NH_3_ in the gas phase. One of the reasons for this is the experimental difficulty of the XANES analysis, which requires high-vacuum conditions that desorb and evacuate the weakly adsorbed NH_3_. In addition, further photoinduced decomposition of NH_3_ eliminated much of the NH_3_ in its gaseous state. Therefore, the N *K*-edge XANES spectra reported here selectively capture the tightly bound form of N, which would exist as NH_4_^+^ in the interlayer space.

## Conclusion and perspectives

The adsorption of NH_3_ on clay minerals caused large N isotopic fractionation. The adsorbed NH_3_ was enriched in ^15^N compared to the initial NH_3_ gas. Although montmorillonite and saponite showed variations in the adsorption ratios and the δ^15^N values due to their interlayer cations and space, dickite, kaolinite, and evacuated samples did not show such variations, and their values settled to certain values. The N *K*-edge XANES spectra of the NH_3_-adsorbed clay minerals revealed that the strongly adsorbed NH_3_ on clay minerals was in the form of ammonium ion (NH_4_^+^), which were chemisorbed on the mineral surface and/or interlayer space. Our study indicated that clay minerals hosted ^15^N-enriched NH_3_ and could have contributed to the formation of ^15^N-enriched organic molecules.

Primordial NH_3_ and N signatures (–NH) on phyllosilicates have been discovered in other solar system objects (e.g., Ceres [42]) and the inner asteroid belt (e.g., carbonaceous asteroid [162173] Ryugu [43, 44]). Recent findings from the analysis of Ryugu samples suggest the important role of N-bearing organic chemical synthesis and diverse molecular profiles on asteroids (e.g., amino acids [5, 45]; amines [6]; nucleobase [46]). Therefore, the successful identification of NH_3_ on the carbonaceous asteroid Ryugu [47] and cometary NH_3_ (67P/Churyumov-Gerasimenko [48]) is of great importance for the N-bearing chemical evolution and ^15^N/^14^N isotopic dynamics in the solar system.

## Data Availability

All data generated or analysed during this study are included in this published article.
